# Inguinal or Para-Aortic Lymph Node Metastasis in Colorectal Cancer: A Narrative Review of Different Treatment Approaches

**DOI:** 10.3390/cancers18203250

**Published:** 2026-10-09

**Authors:** Martin P. Nilsson, Jakob Eberhard, Anders Johnsson, Henrik Jutesten, Marie-Louise Lydrup, Pamela Buchwald

**Affiliations:** 1 Department of Hematology, Oncology and Radiation Physics, Skåne University Hospital, 221 85 Lund, Sweden; 2 Department of Surgery, Skåne University Hospital, 205 02 Malmö, Sweden

**Keywords:** colorectal cancer, inguinal, para-aortic, curative, surgery, radiotherapy

## Abstract

**Background:** Recent studies have indicated that the treatment of inguinal lymph node metastasis (ILNM) and para-aortic lymph node metastasis (PALNM) in colorectal cancer could be curative. However, the optimal treatment approach remains unclear. **Methods:** A systematic search and a narrative review were undertaken to identify manuscripts reporting at least 10 patients with colorectal cancer treated with curative intent for ILNM or PALNM. Each study cohort was categorized into one of four treatment approaches: (1) surgery without radiotherapy; (2) radiotherapy followed by universal surgery; (3) radiotherapy followed by selective surgery; (4) definitive radiotherapy. **Results and Conclusions:** This review supports the use of curative-intent treatment in selected colorectal cancer patients with ILNM or PALNM. Surgery without radiotherapy is the most extensively studied approach. Lower recurrence rates have been reported in some retrospective series using preoperative radiotherapy followed by universal surgery, at the cost of acceptable additional toxicity. In some small studies, definitive radiotherapy has achieved recurrence rates in the range observed in surgical series, but direct comparisons are lacking. Radiotherapy followed by selective surgery may represent a potential strategy for achieving locoregional control while avoiding surgery in selected patients, but evidence is currently limited to two small studies of ILNM. In related clinical settings, such as nonoperative management of primary rectal tumors and lateral lymph node metastasis, treatment has shifted over the past decade toward radiotherapy followed by selective surgery. It is possible that a similar evolution will occur for ILNM and PALNM as well, but stronger evidence is required before firm recommendations can be made. Systemic therapy was not the focus of this review but should be considered an essential component for most patients, in addition to radiotherapy and surgery.

## Simple Summary

This review compared different curative-intent treatment strategies for colorectal cancer patients with inguinal or para-aortic lymph node metastasis. Surgery without radiotherapy remains the most established approach. An alternative strategy is to administer radiotherapy first and reserve surgery for patients whose cancer does not respond adequately to radiotherapy. This approach, known as radiotherapy followed by selective surgery, may potentially improve cancer control while avoiding unnecessary surgical morbidity. It has become increasingly used in similar oncological settings over the past decade. However, for colorectal cancer patients with inguinal or para-aortic lymph node metastasis, this strategy has so far only been evaluated in two small studies, both limited to inguinal lymph node metastases. More research is therefore required before clear treatment recommendations can be made.

## 1. Introduction

Inguinal lymph node metastasis (ILNM) and para-aortic lymph node metastasis (PALNM) are found in <1% and 1–2% of colorectal cancer patients, respectively [1,2]. Based on poor survival in historical series, both ILNM and PALNM have been classified as distant metastasis [3,4]. However, recent studies have suggested that selected patients with ILNM or PALNM can achieve long-term disease control and cure. Accordingly, these two clinical scenarios may be considered together as “non-pelvic lymph node metastasis potentially amenable for curative treatment”.

For rectal cancers extending below the dentate line, the inguinal lymph nodes can represent the first echelon nodal basin. Consistent with this, contemporary series of ILNM in low rectal cancer report survival outcomes more in line with regional lymph node metastasis than with distant metastasis [2]. In contrast, for both colon and rectal cancers, the para-aortic lymph nodes typically constitute second- or third-tier stations, following the mesocolic, the mesorectal, or the lateral lymph node basins. Systematic reviews suggest that para-aortic lymph node dissection (PALND) is associated with improved survival compared to non-surgical management in patients with PALNM [1,5,6]. However, most patients who did not undergo surgery in these reviews were treated with palliative intent [7,8,9,10,11,12,13,14]. Thus, the findings primarily indicate that curative-intent treatment with surgery is superior to a palliative-intent treatment with chemotherapy, rather than establishing the optimal curative strategy.

Importantly, surgery is not the only definitive treatment option for lymph node metastasis in colorectal cancer. In principle, four distinct curative approaches can be considered:

1. Surgery without radiotherapy (RT).
2. RT followed by universal surgery: all patients undergo surgery, irrespective of the response to RT.
3. RT followed by selective surgery: the response to RT determines if surgery is needed or not.
4. Definitive RT: surgery is not planned but might be a salvage option at recurrence.

Previous studies on colorectal cancer with ILNM or PALNM have largely investigated these four treatment strategies separately. Using the format of a narrative review, we therefore aimed to synthesize the available evidence and compare the different approaches. Of note, most patients should also receive chemotherapy (with or without targeted therapy) as part of a curative treatment strategy, given the high risk of subsequent distant relapse. However, because this review focuses on definitive treatment modalities, systemic therapies are discussed only briefly.

## 2. Methods

Eligibility criteria were defined to guide study selection for this review.

Inclusion criteria (all):

1. Published in English in peer-reviewed journals between 1 January 2000 and 31 December 2025.
2. Included ≥ 10 patients with colorectal adenocarcinoma and ILNM or PALNM treated with curative intent.
3. Provided sufficient details to classify the treatment approach.
4. Reported recurrence or survival outcomes.

Exclusion criteria (any):

1. Outcomes for patients with ILNM or PALNM could not be distinguished.
2. Overlapping cohorts were identified; in such cases, only the largest dataset was retained.

A search in the PubMed database conducted on March 1 2026 using the terms (“colorectal cancer” OR “colorectal carcinoma” OR “rectal cancer” OR “rectal carcinoma”) AND (“para-aortic” OR “paraaortic” OR “retroperitoneal” OR “inguinal”) yielded 729 results. All titles were screened by a clinical oncologist (MPN), and abstracts or full texts were reviewed to evaluate eligibility. Reference lists were also examined to identify additional relevant studies. For PALNM, approximately 30 studies evaluating surgery without RT were identified. Rather than including all, a comprehensive review by Fadel et al. covering 19 studies [15], together with the largest additional series by Ito et al. [16], were deemed representative and were therefore selected. In total, seven studies on ILNM and eight studies on PALNM were included in this narrative review [15,16,17,18,19,20,21,22,23,24,25,26,27,28,29]. All the studies were retrospective.

Because the studies were heterogeneous with respect to patient selection and treatment, we constructed the following classification system:

- If >70% of the patients in a study cohort were treated according to a specific treatment approach, it was denoted the “general treatment approach” of that study cohort. Four general treatment approaches were identified:
  (1) Surgery without RT;
  (2) RT followed by universal surgery;
  (3) RT followed by selective surgery;
  (4) Definitive RT.

- The oncological treatment was classified as “intensified” if >50% of the patients in the study cohort received any of the following:
  (a) Neoadjuvant chemotherapy either before or after RT;
  (b) Radiation dose ≥55 Gy combined with concomitant chemotherapy;
  (c) Radiation dose ≥60 Gy without concomitant chemotherapy.

In study cohorts in which one treatment modality was intensified but another modality was de-intensified, an individual assessment was made. For example, in the photon group of the manuscript by Lee et al. [29], 60% of the patients had neoadjuvant chemotherapy (intensification) but only 10% received concomitant chemotherapy (de-intensification). Taken together, the oncological treatment of this cohort was therefore not classified as intensified.

The narrative review format was chosen instead of a systematic review to allow for a broader and more clinically oriented summary of the topic. No formal risk-of-bias assessment was undertaken, and the protocol was not preregistered.

## 3. Results

### 3.1. Inguinal Lymph Node Metastasis (ILNM)

#### 3.1.1. Surgery Without RT for ILNM

Three studies were identified, reporting similar rates of inguinal recurrence (12–15%), 5-year recurrence-free survival (19–33%), and 5-year overall survival (OS; 39–55%) (Table 1 and Table 2) [17,18,19]. In the largest study, Ito et al. included 107 rectal cancer patients with pathologically confirmed ILNM who had undergone inguinal lymph node dissection (ILND) at 20 centers in Japan. Concomitant distant organ metastasis (DOM) was present in 26%. The general treatment approach was surgery without RT with only 4 patients receiving preoperative RT and 16 neoadjuvant chemotherapy. Five-year relapse-free survival was 33%, and five-year OS was 39%. Postoperative inguinal complications of Clavien–Dindo grade III or higher were observed in one patient (0.9%; an inguinal abscess) [18].

#### 3.1.2. RT Followed by Universal Surgery for ILNM

Two studies were found (Table 1 and Table 2) [20,21]. Hasegawa et al. included 15 patients with ILNM (defined by radiological criteria; Table 1). Following RT with concomitant chemotherapy (chemoradiotherapy, CRT) in 12 patients and neoadjuvant chemotherapy in the other 3, all underwent ILND. Tumor cells were found in 4 inguinal surgical specimens; in the other 11, no tumor cells were found, either as a consequence of false-positive radiology or complete response to CRT. No inguinal recurrences were seen. Seven patients (47%) developed postoperative inguinal seroma [20].

Abd El Aziz et al. analyzed 24 patients with ILNM. The median OS was longer for patients with isolated synchronous ILNM than for patients with concomitant DOM (75 vs. 18 months; *p* = 0.09). The authors concluded that synchronous ILNM appeared to represent a distinct subgroup of rectal cancer patients and that treatment with curative intention should be considered for them [21].

#### 3.1.3. RT Followed by Selective Surgery for ILNM

Two studies were identified (Table 1 and Table 2) [22,23]. In the first study, 19 patients with ILNM (defined by radiological criteria; Table 1) were treated with CRT followed by selective surgery. ILND was omitted if lymph node sizes decreased to <10 mm or showed a marked reduction in SUV max on PET-CT after CRT. Seven of nineteen patients underwent ILND; all had residual tumor in the inguinal surgical specimen and one developed inguinal recurrence during follow-up. None of the 12 patients who did not undergo ILND experienced inguinal recurrence. Persistent lower-limb lymphedema occurred in 29% of patients treated with RT followed by ILND, compared to 13% of patients treated with ILND alone, and 0% of patients receiving RT only [22].

In the second study, 16 patients with isolated synchronous ILNM received neoadjuvant chemotherapy combined with dose-escalated CRT. Metastatic lymph nodes were biopsy-confirmed before treatment. Only patients with persistently suspicious inguinal nodes (short axis diameter ≥ 8 mm or malignant features on MRI; Table 1) on restaging after oncological therapy proceeded to ILND. Five patients fulfilled radiological criteria and underwent ILND, of whom two had residual malignant cells in the inguinal specimen. Among the 11 patients who avoided ILND, 3 developed distant metastasis during follow-up, but none recurred in the inguinal region, suggesting a durable complete response. All patients underwent resection of the primary tumor; exclusion of nonoperated patients may have introduced selection bias. Toxicities were mainly grade I-II, with no life-threatening or disabling events [23].

#### 3.1.4. Definitive RT for ILNM

No studies on definitive RT for ILNM were found.

### 3.2. Para-Aortic Lymph Node Metastasis (PALNM)

#### 3.2.1. Surgery Without RT for PALNM

The systematic review by Fadel et al. included 19 studies comprising 541 patients with PALNM from colon cancer (76%) or rectal cancer (24%), presenting synchronously (67%) or metachronously (33%) (Table 3 and Table 4) [15]. Most studies originated from Japan, where PALNM has traditionally been treated surgically. RT was rarely used, whereas 92% of patients received chemotherapy. Across studies, the median para-aortic recurrence rate was 19% (range 0–55%), the median 5-year disease-free survival was 24% (range 10–61%), and the median 5-year OS was 47% (range 15–88%). Postoperative Clavien–Dindo grade III or higher complications occurred in 13% of patients. The authors concluded that PALND is a feasible option with acceptable morbidity and possible oncological benefit for patients with PALNM [15].

In the largest study to date, Ito et al. included 133 patients with colon cancer (41%) or rectal cancer (59%) who underwent PALND across 36 centers in Japan; all had histopathologically confirmed PALNM. Preoperative RT was rarely used (2 patients), and 76% received neoadjuvant or adjuvant chemotherapy. Among 96 patients with R0 resection, the 5-year cumulative incidence of recurrence was 76%. Recurrences most commonly involved the para-aortic lymph nodes (33%) and lungs (32%), followed by other distant lymph nodes (17%) and the liver (13%). On multivariable analysis, rectal or left-sided primary tumors, concomitant distant metastasis, ≥3 metastatic para-aortic lymph nodes, and a low institutional volume (< 10 cases) were associated with worse recurrence-free survival. The results indicated that PALND for PALNM should be centralized to high-volume centers [16].

#### 3.2.2. RT Followed by Universal Surgery for PALNM

Two studies evaluated RT followed by universal PALND [25,26], and one assessed the addition of preoperative RT [22] (Table 3 and Table 4). Although cross-study comparisons should be interpreted cautiously, reported para-aortic recurrence rates (6–20%) were lower than in surgery-only series (19–58%), suggesting that preoperative RT may improve local control without a clear impact on 5-year OS (Table 4).

In the study by Houbiers et al., patients received neoadjuvant chemotherapy followed by PALND (*n* = 24) or preoperative RT plus PALND (*n* = 30). Patients progressing during neoadjuvant chemotherapy were excluded, introducing potential selection bias. Preoperative RT reduced lymph node recurrence (20 vs. 58%; *p* = 0.005) but did not improve 5-year OS. Preoperative RT was associated with a higher rate of postoperative chylous leakage (21 vs. 4%; *p* = 0.19) without increasing severe postoperative complications [24].

At the Mayo Clinic, a trimodality approach including preoperative CRT, surgery, and intraoperative RT, has been used for PALNM. In a consecutive retrospective cohort of 65 patients with colorectal cancer and isolated abdominal nodal metastasis (85% para-aortic), Johnson et al. reported a low para-aortic recurrence rate (6%) but frequent distant relapses, resulting in a 5-year progression-free survival of approximately 30%. The authors concluded that this strategy yields cure rates comparable to metastasectomy for liver and lung oligometastases [25].

#### 3.2.3. RT Followed by Selective Surgery for PALNM

No studies on RT followed by selective surgery for PALNM were found.

#### 3.2.4. Definitive RT for PALNM

Three studies were identified (Table 3 and Table 4) [27,28,29]. In the first, 22 patients with metachronous isolated PALNM were treated with dose-escalated CRT (median 63 Gy). The diagnosis of PALNM was based on strict radiological criteria (Table 3), without mandatory biopsy. During follow-up, para-aortic recurrence occurred in 32% of the patients, and the 5-year recurrence-free survival was 26%. Toxicity was limited, and CRT was considered an effective salvage option that may avoid surgical morbidity [27].

In the second study, Shu et al. included 68 patients treated with neoadjuvant chemotherapy and dose-escalated RT. Outcomes were markedly better in patients with isolated PALNM than in patients with DOM (5-year OS 45 vs. 0%). However, RT provided symptom relief of pain and urinary obstruction in patients with disseminated disease [28].

The third study compared photon RT versus carbon ion RT. Para-aortic control rates were relatively low in both groups (5-year local control 43 and 64%, respectively), possibly related to limited RT field coverage. Late toxicity, reported only in the photon group, consisted of mild gastrointestinal symptoms [29].

## 4. Discussion

This narrative review summarizes outcomes after curative-intent treatment of ILNM and PALNM in colorectal cancer. The results suggest that survival among patients with ILNM is comparable to that observed in patients with locoregional nodal disease, whereas outcomes in patients with PALNM appear similar to those reported for isolated liver or lung metastasis. Thus, the available evidence supports treating selected patients with ILNM or PALNM from colorectal cancer with curative intent.

The primary aim was to categorize and compare contemporary curative-intent strategies: (1) surgery without RT; (2) RT followed by universal surgery; (3) RT followed by selective surgery; (4) definitive RT. Previous studies have largely evaluated these approaches in isolation or compared them only with palliative treatments.

Surgical management of ILNM and PALNM in colorectal cancer is currently supported almost exclusively by retrospective case series. Even in the largest PALND series, R0 resection was followed by recurrence in more than two thirds of patients, and 5-year OS seldom exceeded 50%, indicating that surgery alone rarely eradicates systemic disease and may primarily improve regional control [15,16]. For ILNM, long-term survival is certainly achievable, but outcomes after surgery alone remain modest and morbidity, particularly chronic lymphedema and wound healing problems, is non negligible [17,18,19]. Surgical technique, extent of lymphadenectomy, and institutional volume are rarely standardized or comprehensively reported. Nevertheless, available data suggest that outcomes are strongly volume-dependent, which limits generalizability and obscures the true benefit of optimal surgery [7,13,21].

When RT is used to avoid surgery, two treatment-related factors appear particularly important: the extent of the RT field, and the intensity of the oncological treatment. The RT field should encompass not only pathologic lymph nodes, but also adjacent non-enlarged lymph nodes at risk of harboring micrometastasis. For a superficial ILNM, it could be appropriate to include the deep inguinal nodes, ipsilateral external iliac nodes, and contralateral inguinal nodes. For PALNM, an adequate craniocaudal margin is crucial. Lee et al. analyzed 52 patients with isolated PALNM treated with definitive RT [9]. In many cases, the RT field encompassed only a limited margin around the pathologic lymph nodes rather than the entire para-aortic region. Para-aortic recurrences were common (62%), with more than half occurring outside the RT field. Yeo et al. and Shu et al. used larger margins and reported lower para-aortic recurrence rates (30–32%) [27,28]. The importance of a generous para-aortic margin is also supported by data from other malignancies, including anal squamous cell carcinoma [30]. The NOAC anal cancer guidelines recommend extending the cranial border of the RT field to the left renal vein or 3 cm above the most cranially located PALNM, whichever is higher [31]. In the absence of colorectal cancer-specific RT contouring guidelines for PALNM, the NOAC anal cancer guidelines may provide useful guidance.

The standard preoperative treatment for rectal cancer has long been 50 Gy CRT [32]. Pathological complete response following 50 Gy CRT is achieved in approximately 10–15% of patients with locally advanced rectal cancer [33]. More recent randomized trials have investigated treatment intensification either by adding neoadjuvant chemotherapy or escalating RT dose, reporting primary tumor complete response rates around 30–50% [33,34,35]. However, the chance of complete response is higher in lymph node metastasis than in primary tumors [32]. In the study by Chen et al., 16 patients with biopsy-confirmed ILNM were treated with neoadjuvant chemotherapy combined with 50 Gy CRT to the primary tumor and 58 Gy to pathologic inguinal lymph nodes. Complete response was achieved in 19% of primary tumors compared with 88% of ILNM. Although the small sample size precludes direct attribution of this difference to RT dose, these findings illustrate both the potentially greater radiosensitivity of nodal metastases compared with primary tumors and the feasibility of dose escalation to involved lymph nodes [23].

It could be concluded that achieving high para-aortic and inguinal tumor control with RT alone likely requires both extended RT fields and treatment intensification. Whether such an approach can be delivered with acceptable toxicity remains an important question. Based on this review, the preliminary answer appears to be yes, but the evidence is limited by small study sizes and requires further confirmation. A key concern is timing of response assessment. Because RT response may take several months to fully manifest, patients considered for ILND or PALND after incomplete response will undergo delayed surgery. Compared with preoperative RT with universal surgery, where the surgical procedure is planned 1–2 months after the end of RT, delayed surgery performed 3–6 months after RT may be more challenging due to radiation-induced fibrosis.

Overall, surgery without RT remains the most established curative-intent treatment approach for ILNM or PALNM in colorectal cancer. Lower recurrence rates have been reported in some retrospective series using preoperative radiotherapy followed by universal surgery, at the cost of acceptable additional toxicity. In some small studies, intensified definitive RT with adequate RT field coverage has achieved recurrence rates in the range observed in surgical series, but direct comparisons are lacking. RT followed by selective surgery is a potentially attractive strategy because it may combine durable disease control with avoidance of surgery in patients achieving a complete response. However, this approach is currently supported by only two small studies of ILNM [22,23], and the available evidence is insufficient to draw firm conclusions.

Evidence from related clinical settings may provide some support for this treatment paradigm. In rectal cancer, treatment has shifted over the past decade toward RT followed by selective surgery, both for the primary tumor and for lateral lymph node metastasis. Growing evidence supports nonoperative management as a safe alternative to abdominoperineal resection in patients achieving a complete response after RT. Similarly, data from the Lateral Node Study Consortium have shown that lateral lymph node dissection can be safely omitted when nodes regress to <4 mm after CRT [36]. For ILNM and PALNM, optimal radiological response criteria remain undefined, which is currently a major limitation. Shiratori et al. omitted inguinal surgery when lymph nodes regressed to <10 mm or showed a marked reduction in SUV max on PET-CT after RT [22], whereas Chen et al. avoided inguinal surgery when post-RT nodes measured <8 mm and lacked irregular borders or mixed signal intensity on MRI [23]. In these small cohorts, none of the 23 patients fulfilling the criteria developed inguinal recurrence during follow-up. Defining optimal radiological response criteria for ILNM and PALNM in larger cohorts should be a priority in future studies.

Importantly, PALNM should not be regarded as a biologically homogeneous entity. The timing of nodal metastasis, number and distribution of involved lymph nodes, presence of concomitant organ metastases, primary tumor location, RAS/BRAF and MMR/MSI status, and response to systemic therapy are likely to define biologically distinct subgroups with different prospects for cure. These differences might, in turn, provide a rationale for tailoring treatment sequencing and dose intensity to the underlying biology of the disease.

This review is limited by the retrospective nature of the available data, small patient numbers, and the absence of a formal risk-of-bias assessment. All available studies are susceptible to substantial selection bias, as patients offered curative-intent treatments typically have favorable biology, limited disease burden, and good performance status. Furthermore, lymph node metastases were frequently diagnosed on the basis of imaging rather than pathological confirmation, particularly for para-aortic nodes, for which biopsy is technically challenging. This introduces the possibility of false-positive findings and complicates comparisons between studies. For example, in a surgical series, the positive predictive value of a 10 mm threshold for PALNM was only 54% [37]. Although MRI, PET-CT, and tumor characteristics may improve diagnostic accuracy, radiological overestimation of nodal disease remains a concern and may inflate survival estimates for non-surgical approaches.

## 5. Conclusions

This review supports treating selected patients with ILNM or PALNM from colorectal cancer with curative intent. Surgery without RT is the most studied treatment approach. RT followed by selective surgery may represent a potential strategy for achieving locoregional control while avoiding surgery in selected patients, but evidence is currently limited to two small studies of ILNM. In related clinical settings such as nonoperative management of primary rectal tumors and lateral lymph node metastasis, treatment has shifted over the past decade toward RT followed by selective surgery. It is possible that a similar evolution will occur also for ILNM and PALNM, but stronger evidence is required before any firm recommendations can be made. Systemic therapy was not the focus of this review but should be considered essential for most patients, in addition to RT and surgery.

## Figures and Tables

**Table 1:** Overview of studies on curative-intent treatment of ILNM in rectal cancer.

First Author	Country	Year of Treatment	Number of Patients	Concomitant DOM (%)	ILNM Verification	Radiological Criteria for Pathological Inguinal Lymph Nodes
Tanabe (2019) [17]	Japan	1986–2017	31	6	P	n/a
Ito (2024(1)) [18]	Japan	1997–2011	107	26	P	n/a
Hagemans (2019) ^a^ [19]	Netherlands	2005–2017	19	16	P	n/a
Hasegawa (2022) [20]	Japan	2005–2019	15	0	R	No strict definition; however, a SAD ≥ 10 mm, an abnormal shape, and an abnormal accumulation of FDG were defined as clinically positive for metastasis.
Abd El Aziz (2022) [21]	USA	2002–2020	24	33	n/a	n/a
Shiratori (2020) ^b^ [22]	Japan	2011–2019	19	0	R	*Before RT*: SAD > 10 mm, or a high SUV max on PET. *After RT*: ILND was omitted when the lymph node sizes decreased to <10 mm or the SUV max remarkably decreased.
Chen (2021) [23]	China	2017–2019	16	0	P	*Before RT*: SAD ≥ 8 mm, positive on diffusion-weighted MRI, irregular borders on MRI or CT, or hot spots on PET. *After RT*: SAD ≥ 8 mm on CT or MRI, or irregular borders and mixed signal intensity on MRI.

Abbreviations: CT, computed tomography; DOM, distant organ metastasis; FDG, fluorodeoxyglocose; ILND, inguinal lymph node dissection; ILNM, inguinal lymph node metastasis; MRI, magnetic resonance imaging; P, pathology; PET, positron emission tomography; R, radiology; RT, radiotherapy; SAD, short axis diameter; SUV, standardized uptake value. ^a^ Only patients treated with curative intent from this publication. ^b^ Only patients treated from 2011 and onwards from this publication, when the treatment approach was changed.

**Table 2:** General treatment approach and outcomes in studies on curative-intent treatment of ILNM in rectal cancer.

First Author	General Treatment Approach for ILNM	RT Dose ^a^ (Median)	Intensified Oncological Treatment ^b^	Inguinal Recurrence ^c^ (%)	5-Year RFS ^d^ (%)	5-Year OS (%)
Tanabe (2019) [17]	Surgery without RT	n/a	No	13	19 ^e^	55
Ito (2024(1)) [18]	Surgery without RT	n/a	No	15 ^f^	33 ^g^	39
Hagemans (2019) [19]	Surgery without RT ^h^	n/a	No	12 ^i^	26 ^e^	34 ^j^
Hasegawa (2022) [20]	RT → Universal surgery ^k^	45–50.4 Gy	No	0	64 ^l^	78
Abd El Aziz (2022) [21]	RT → Universal surgery	50.4 Gy	Yes	19	30 ^m,n^	53 ^o^
Shiratori (2020) [22]	RT → Selective surgery	50.4 Gy	No	5	35 ^p,l^	56 ^l^
Chen (2021) [23]	RT → Selective surgery	58 Gy	Yes	0	81 ^q^ (3-year)	100 ^r^ (3-year)

Abbreviations: ILNM, inguinal lymph node metastasis; OS, overall survival; RFS, recurrence-free survival. ^a^ Dose to ILNM. ^b^ For definition, see the Section 2. ^c^ Ipsilateral or contralateral. ^d^ Includes recurrence-free survival, relapse-free survival, progression-free survival, disease-free survival, and proportion of patients without recurrence at last follow-up. ^e^ Proportion of patients without recurrence at last follow-up. ^f^ Not reported in this publication, but reported in Sato et al. (Colorectal Dis 2022, 24(10):1150–1163) on the same cohort. ^g^ Recurrence-free survival. ^h^ 17 of 19 patients received preoperative RT, but the inguinal region was included in the target volume only for 2 patients. ^i^ 2 of 17 patients who underwent inguinal surgery. ^j^ 34% for all 19 patients. For patients with isolated ILNM, the 5-year OS was 52%. ^k^ Not explicitly stated that the inguinal region was included in the target volume. ^l^ Relapse-free survival. ^m^ 30% for patients with isolated synchronous ILNM and 0% for patients with DOM. Not reported for the entire cohort. ^n^ Progression-free survival, visually estimated from the Kaplan–Meier curve. ^o^ 53% for patients with isolated synchronous ILNM and 21% for patients with DOM. Not reported for the entire cohort. ^p^ 56% for patients who underwent inguinal surgery. Not reported for patients who had a complete response following RT. ^q^ Disease-free survival. ^r^ With only 16 patients, the confidence interval is wide.

**Table 3:** Overview of studies on curative-intent treatment of PALNM in colorectal cancer.

First Author	Country	Year of Treatment	Number of Patients	Concomitant DOM (%)	PALNM Verification	Radiological Criteria for Pathologic Para-Aortic Lymph Nodes
Fadel (2023) ^a^ [15]	Mixed	1984–2018	541	n/a	P/R	The most commonly used criteria among the included studies were SAD > 8 mm, or PET-positive.
Ito (2024(2)) [16]	Japan	2011–2015	133	27	P	n/a
Houbiers (2025) [24]	France	2000–2023	24 ^b^	46	P	SAD ≥ 10 mm (especially with irregular outer border, round shape, or heterogeneous internal density), or hypermetabolic on PET.
	France	2000–2023	30 ^c^	40	P	
Johnson (2018) [25]	USA	1994–2016	65	0	P/R	n/a
Bhutani (2024) [26]	USA	2007–2020	74	14	R	n/a
Yeo (2010) [27]	Korea	2002–2007	22	0	R	Any of the following: (1) growing retroperitoneal LN ≥ 1 cm on two consecutive CTs performed at 3-month intervals; (2) retroperitoneal LN ≥ 2 cm on CT; (3) two consecutive elevations over the normal CEA level at 3-month intervals and retroperitoneal LN ≥ 1 cm on CT; or (4) two consecutive elevations over the normal CEA level at 3-month intervals and multiple retroperitoneal LNs ≥ 0.5 cm on CT.
Shu (2020) [28]	China	2009–2018	40 ^d^	0	R	n/a
	China	2009–2018	28 ^e^	100	R	
Lee (2025) [29]	Korea	2006–2023	63 ^f^	0	n/a	n/a
	Japan	2006–2023	53 ^g^	0	n/a	

Abbreviations: CEA, carcinoembryonic antigen; CT, computed tomography; DOM, distant organ metastasis; LN, lymph node; P, pathology; PALNM, para-aortic lymph node metastasis; PET, positron emission tomography; R, radiology; SAD, short axis diameter. ^a^ Review of 19 studies. ^b^ Surgery group from this publication. ^c^ RT followed by surgery group from this publication. ^d^ Isolated PALNM group from this publication. ^e^ Concomitant DOM group from this publication. ^f^ Photon group from this publication. ^g^ Carbon ion group from this publication.

**Table 4:** General treatment approach and outcomes in studies on curative-intent treatment of PALNM in colorectal cancer.

First Author	General Treatment Approach for PALNM	RT Dose ^a^ (Median)	Intensified Oncological Treatment ^b^	Para-Aortic Recurrence (%)	5-Year RFS ^c^ (%)	5-Year OS (%)
Fadel (2023) ^d^ [15]	Surgery without RT	n/a	No	19 ^e^	24 ^f^	47
Ito (2024(2)) [16]	Surgery without RT	n/a	No	33 ^g^	21 ^g^	41
Houbiers (2025) [24]	Surgery without RT ^h^	n/a	No	58 ^i^	15 ^j^	60 ^j^
	RT → Universal surgery ^k^	50 Gy	Yes	20 ^i^	35 ^j^	60 ^j^
Johnson (2018) [25]	RT → Universal surgery	80 Gy ^l^	Yes	6	30 ^j,m^	45
Bhutani (2024) [26]	RT → Universal surgery ^n^	n/a	No	n/a	15/70 ^j,o^	44
Yeo (2010) [27]	Definitive RT	63 Gy	Yes	32	26	36
Shu (2020) [28]	Definitive RT ^p^	60 Gy	Yes	30 ^q^	30 ^j,m^	45 ^j^
	Definitive RT ^r^	60 Gy	Yes	n/a	0 ^j,m^	0
Lee (2025) [29]	Definitive RT ^s^	56 Gy	No ^t^	58 ^u^	5 ^j,m^	40 ^j^
	Definitive RT ^v^	71 Gy ^x^	Yes	36 ^y^	15 ^j,m^	30 ^j^

Abbreviations: OS, overall survival; PALNM, para-aortic lymph node metastasis; RFS, recurrence-free survival; RT, radiotherapy. ^a^ Dose to PALNM. ^b^ For definition, see the Methods section. ^c^ Includes recurrence-free survival, disease-free survival, and progression-free survival. ^d^ Review of 19 studies. ^e^ Median (range 0–55%) among 15 studies with para-aortic recurrence reported. ^f^ Disease-free survival. ^g^ Only reported for 96 patients with R0 resection. ^h^ Surgery group from this publication. ^i^ Includes lymph node recurrence in the mediastinum, para-aortic, and below the aortic bifurcation (para-aortic recurrence not reported separately). ^j^ Visually estimated from the Kaplan–Meier curve. ^k^ RT followed by surgery group from this publication. ^l^ Median external beam RT dose 50.4 Gy, median intraoperative RT dose 12.5 Gy. In total (α⁄β = 5), EQD2 = 80 Gy. ^m^ Progression-free survival. ^n^ 60 patients received preoperative chemotherapy with or without RT (not specified how many received RT). ^o^ 15% for patients with positive PALN and 70% for patients with negative PALN in surgical specimen. ^p^ Isolated PALNM group from this publication. ^q^ Based on 3-year local control 70%. ^r^ Concomitant DOM group from this publication. ^s^ Photon group from this publication. ^t^ 60% neoadjuvant chemotherapy but only 10% concomitant chemotherapy and therefore assessed non-intensified. ^u^ Based on 5-year local control 42%. ^v^ Carbon ion group from this publication. ^x^ Median 52.8 Gy[RBE]/12 fractions (α⁄β = 5), EQD2 = 71 Gy. RBE of carbon ions was set to 3.0. ^y^ Based on 5-year local control 64%.

## Data Availability

No new data were created or analyzed in this study. Data sharing is not applicable to this article.

## Abbreviations

The following abbreviations are used in this manuscript:

CRT	chemoradiotherapy
DOM	distant organ metastasis
EQD2	equivalent dose in 2 Gy fractions
ILND	inguinal lymph node dissection
ILNM	inguinal lymph node metastasis
NOAC	Nordic Anal Cancer Group
OS	overall survival
PALND	para-aortic lymph node dissection
PALNM	para-aortic lymph node metastasis
PET	positron emission tomography
RT	radiotherapy
SAD	short axis diameter
SUV	standardized uptake value

## References

[1] Chen M.Z., Tay Y.K., Prabhakaran S., Kong J.C. (2023). The management of clinically suspicious para-aortic lymph node metastasis in colorectal cancer: A systematic review. Asia Pac. J. Clin. Oncol..

[2] Wyatt J., Powell S.G., Ahmed S., Arthur J., Altaf K., Ahmed S., Javed M.A. (2023). Inguinal lymph node metastases from rectal adenocarcinoma: A systematic review. Tech. Coloproctol..

[3] Graham R.A., Hohn D.C. (1990). Management of inguinal lymph node metastases from adenocarcinoma of the rectum. Dis. Colon. Rectum.

[4] Luna-Pérez P., Corral P., Labastida S., Rodríguez-Coria D., Delgado S. (1999). Inguinal lymph node metastases from rectal adenocarcinoma. J. Surg. Oncol..

[5] Onafowokan O.O., Redfern J., Patel A., Satyadas T., Baltatzis M. (2023). Outcomes of metachronous para-aortic lymphadenectomy in colorectal cancer: A systematic review of the literature. Langenbeck’s Arch. Surg..

[6] Wang R.C., Wang J.Q., Zhou X.Y., Zhong C.L., Chen J.X., Chen J.S. (2023). Survival benefits of para-aortic lymphadenectomy in colorectal cancer with clinically suspected para-aortic lymph node metastasis: A meta-analysis and systematic review. World J. Surg. Oncol..

[7] Choi P.W., Kim H.C., Kim A.Y., Jung S.H., Yu C.S., Kim J.C. (2010). Extensive lymphadenectomy in colorectal cancer with isolated para-aortic lymph node metastasis below the level of renal vessels. J. Surg. Oncol..

[8] Kim Y.I., Park I.J., Park J.H., Kim T.W., Ro J.S., Lim S.B., Yu C.S., Kim J.C. (2020). Management of isolated para-aortic lymph node recurrence after surgery for colorectal cancer. Ann. Surg. Treat. Res..

[9] Lee S.C., Kim H.C., Lee W.Y., Yun S.H., Cho Y.B., Huh J.W., Park Y.A., Shin J.K. (2021). Effect of lymphadenectomy in colorectal cancer with isolated synchronous para-aortic lymph node metastasis. Colorectal Dis..

[10] Min B.S., Kim N.K., Sohn S.K., Cho C.H., Lee K.Y., Baik S.H. (2008). Isolated paraaortic lymph-node recurrence after the curative resection of colorectal carcinoma. J. Surg. Oncol..

[11] Nozawa H., Kawai K., Sasaki K., Emoto S., Abe S., Sonoda H., Murono K., Kishikawa J., Nagai Y., Yokoyama Y. (2022). Predictive factors of survival of colorectal cancer patients after para-aortic lymph node metastasis. Int. J. Clin. Oncol..

[12] Ogura A., Akiyoshi T., Takatsu Y., Nagata J., Nagasaki T., Konishi T., Fujimoto Y., Nagayama S., Fukunaga Y., Ueno M. (2017). The significance of extended lymphadenectomy for colorectal cancer with isolated synchronous extraregional lymph node metastasis. Asian J. Surg..

[13] Shibata D., Paty P.B., Guillem J.G., Wong W.D., Cohen A.M. (2002). Surgical management of isolated retroperitoneal recurrences of colorectal carcinoma. Dis. Colon. Rectum.

[14] Tentes A.A., Mirelis C., Karanikiotis C., Korakianitis O. (2007). Radical lymph node resection of the retroperitoneal area for left-sided colon cancer. Langenbeck’s Arch. Surg..

[15] Fadel M.G., Ahmed M., Pellino G., Rasheed S., Tekkis P., Nicol D., Kontovounisios C., Mayer E. (2023). Retroperitoneal Lymph Node Dissection in Colorectal Cancer with Lymph Node Metastasis: A Systematic Review. Cancers.

[16] Ito S., Kinugasa Y., Yamauchi S., Sato H., Hirakawa A., Ishihara S., Shiomi A., Kanemitsu Y., Suto T., Takahashi H. (2024). Long-term Outcome After Surgical Resection of Para-aortic Lymph Node Metastasis of Colorectal Cancer: A Multicenter Retrospective Study. Dis. Colon. Rectum.

[17] Tanabe T., Shida D., Komukai S., Nakamura Y., Tsukamoto S., Kanemitsu Y. (2019). Long-term outcomes after surgical dissection of inguinal lymph node metastasis from rectal or anal canal adenocarcinoma. BMC Cancer.

[18] Ito S., Tsukamoto S., Kagawa H., Kanemitsu Y., Hiro J., Kawai K., Nozawa H., Takii Y., Yamaguchi T., Akagi Y. (2024). Short- and long-term outcomes of surgical treatment for inguinal lymph node metastasis in rectal and anal canal adenocarcinoma. Colorectal Dis..

[19] Hagemans J.A.W., Rothbarth J., van Bogerijen G.H.W., van Meerten E., Nuyttens J., Verhoef C., Burger J.W.A. (2019). Treatment of Inguinal Lymph Node Metastases in Patients with Rectal Adenocarcinoma. Ann. Surg. Oncol..

[20] Hasegawa H., Matsuda T., Yamashita K., Sawada R., Harada H., Urakawa N., Goto H., Kanaji S., Oshikiri T., Kakeji Y. (2022). Clinical outcomes of neoadjuvant therapy followed by selective inguinal lymph node dissection and total mesorectal excision for metastasized low rectal cancer. Langenbeck’s Arch. Surg..

[21] Abd El Aziz M.A., McKenna N.P., Jakub J.W., Hallemeier C.L., Kelley S.R., Jin Z., Mathis K.L. (2022). Rectal cancer with synchronous inguinal lymph node metastasis without distant metastasis. A call for further oncological evaluation. Eur. J. Surg. Oncol..

[22] Shiratori H., Nozawa H., Kawai K., Hata K., Tanaka T., Kaneko M., Emoto S., Sonoda H., Ishihara S. (2020). Risk factors and therapeutic significance of inguinal lymph node metastasis in advanced lower rectal cancer. Int. J. Colorectal Dis..

[23] Chen M., Liu S., Xu M., Yi H.C., Liu Y., He F. (2021). Radiation boost for synchronous solitary inguinal lymph node metastasis during neoadjuvant chemoradiotherapy for locally advanced rectal cancer. Discov. Oncol..

[24] Houbiers A., Barbe R., Durand-Labrunie J., Boige V., Smolenschi C., Hollebecque A., Menu Y., Valery M., Ducreux M., Boileve A. (2026). Multimodal Management of Retroperitoneal Lymph Node Metastases in Colorectal Cancer: A Single-Center Retrospective Analysis. Ann. Surg. Oncol..

[25] Johnson B., Jin Z., Haddock M.G., Hallemeier C.L., Martenson J.A., Smoot R.L., Larson D.W., Dozois E.J., Nagorney D.M., Grothey A. (2018). A Curative-Intent Trimodality Approach for Isolated Abdominal Nodal Metastases in Metastatic Colorectal Cancer: Update of a Single-Institutional Experience. Oncologist.

[26] Bhutiani N., Ochiai K., Awiwi M.O., Rodriguez-Bigas M.A., Uppal A., Peacock O., Messick C., White M.G., Skibber J.M., Bednarski B.K. (2024). Para-aortic Lymph Node Dissection for Colorectal Cancer: Predicting Pathologic Lymph Node Positivity and Optimizing Outcomes. Ann. Surg. Oncol..

[27] Yeo S.G., Kim D.Y., Kim T.H., Jung K.H., Hong Y.S., Kim S.Y., Park J.W., Choi H.S., Oh J.H. (2010). Curative chemoradiotherapy for isolated retroperitoneal lymph node recurrence of colorectal cancer. Radiother. Oncol..

[28] Shu P., Ouyang G., Wang F., Zhou J., Shen Y., Li Z., Wang X. (2020). The Role of Radiotherapy in the Treatment of Retroperitoneal Lymph Node Metastases from Colorectal Cancer. Cancer Manag. Res..

[29] Lee J.J.B., Isozaki T., Choi S.H., Takiyama H., Lee J., Chang J.S., Yamada S., Koom W.S. (2025). Outcomes of Carbon-Ion Radiation Therapy Versus Photon Therapy for Isolated Paraortic Lymph Node Recurrence from Colorectal Cancer. Int. J. Radiat. Oncol. Biol. Phys..

[30] Holliday E.B., Lester S.C., Harmsen W.S., Eng C., Haddock M.G., Krishnan S., Das P., Hallemeier C.L. (2018). Extended-Field Chemoradiation Therapy for Definitive Treatment of Anal Canal Squamous Cell Carcinoma Involving the Para-Aortic Lymph Nodes. Int. J. Radiat. Oncol. Biol. Phys..

[31] Nilsson M.P., Undseth C., Albertsson P., Eidem M., Havelund B.M., Johannsson J., Johnsson A., Radu C., Serup-Hansen E., Spindler K.L. (2023). Nordic anal cancer (NOAC) group consensus guidelines for risk-adapted delineation of the elective clinical target volume in anal cancer. Acta Oncol..

[32] Sauer R., Becker H., Hohenberger W., Rödel C., Wittekind C., Fietkau R., Martus P., Tschmelitsch J., Hager E., Hess C.F. (2004). Preoperative versus postoperative chemoradiotherapy for rectal cancer. N. Engl. J. Med..

[33] Moretto R., Boccaccio C., Landi M., Masi G., Cremolini C. (2025). Total neoadjuvant treatment, non-operative management and radiotherapy-free strategies: New approaches for the management of proficient mismatch repair/microsatellite stable locally advanced rectal cancer. A narrative review and evidence-based algorithm. Eur. J. Cancer.

[34] Bahadoer R.R., Dijkstra E.A., van Etten B., Marijnen C.A.M., Putter H., Kranenbarg E.M., Roodvoets A.G.H., Nagtegaal I.D., Beets-Tan R.G.H., Blomqvist L.K. (2021). Short-course radiotherapy followed by chemotherapy before total mesorectal excision (TME) versus preoperative chemoradiotherapy, TME, and optional adjuvant chemotherapy in locally advanced rectal cancer (RAPIDO): A randomised, open-label, phase 3 trial. Lancet Oncol..

[35] Garcia-Aguilar J., Patil S., Gollub M.J., Kim J.K., Yuval J.B., Thompson H.M., Verheij F.S., Omer D.M., Lee M., Dunne R.F. (2022). Organ Preservation in Patients with Rectal Adenocarcinoma Treated with Total Neoadjuvant Therapy. J. Clin. Oncol..

[36] Ogura A., Konishi T., Beets G.L., Cunningham C., Garcia-Aguilar J., Iversen H., Toda S., Lee I.K., Lee H.X., Uehara K. (2019). Lateral Nodal Features on Restaging Magnetic Resonance Imaging Associated with Lateral Local Recurrence in Low Rectal Cancer After Neoadjuvant Chemoradiotherapy or Radiotherapy. JAMA Surg..

[37] Zhou Y., Xie X., Chen X., Tang Q., Cai Z., Zou Y., Yu Z., Chen Y. (2025). Prognostic Impact of Para-Aortic Lymph Node Dissection in Colorectal Cancer Patients Suspected of Para-Aortic Lymph Node Metastasis: A Retrospective Cohort Study. Clin. Colorectal Cancer.

